# Caspase-independent cell death in lung cancer: from mechanisms to clinical applications

**DOI:** 10.1007/s00210-025-04149-0

**Published:** 2025-04-21

**Authors:** Gaurav Gupta, Vijaya Paul Samuel, Rekha M. M., Bindu Rani, Y. Sasikumar, Priya Priyadarshini Nayak, Puneet Sudan, Kavita Goyal, Brian G. Oliver, Amlan Chakraborty, Kamal Dua

**Affiliations:** 1 Centre for Research Impact & Outcome, Chitkara College of Pharmacy, Chitkara University, Rajpura, Punjab 140401 India; 2 Centre of Medical and Bio-Allied Health Sciences Research, Ajman University, Ajman, United Arab Emirates; 3 Department of Anatomy, RAK College of Medicine, RAK Medical and Health Sciences University, Ras Al Khaimah, UAE; 4 Department of Chemistry and Biochemistry, School of Sciences, JAIN (Deemed to Be University), Bangalore, Karnataka India; 5 Department of Medicine, National Institute of Medical Sciences, NIMS University Rajasthan, Jaipur, India; 6 Department of CHEMISTRY, Sathyabama Institute of Science and Technology, Chennai, Tamil Nadu India; 7 Department of Medical Oncology IMS and SUM Hospital, Siksha ‘O’ Anusandhan (Deemed to Be University), Bhubaneswar, Odisha 751003 India; 8 Department of Pharmacy, Chandigarh Pharmacy College, Chandigarh Group of Colleges-Jhanjeri, Mohali, 140307 Punjab India; 9 Department of Biotechnology, Graphic Era (Deemed to Be University), Clement Town, Dehradun, 248002 India; 10 Woolcock Institute of Medical Research, Macquarie University, Sydney, NSW Australia; 11 School of Life Sciences, University of Technology Sydney, Ultimo, NSW 2007 Australia; 12 Faculty of Biology, Medicine and Health, The University of Manchester, Oxford Road, Manchester, M13 9PL UK; 13 Cardiovascular Disease Program, Biomedicine Discovery Institute and Department of Pharmacology, Monash University, Clayton, VIC 3800 Australia; 14 Discipline of Pharmacy, Graduate School of Health, University of Technology Sydney, Ultimo, NSW 2007 Australia; 15 Faculty of Health, Australian Research Centre in Complementary and Integrative Medicine, University of Technology Sydney, Ultimo, NSW 2007 Australia

**Keywords:** Caspase-independent cell death, Lung cancer, Ferroptosis, Necroptosis, Mitochondrial dysfunction, Autophagy

## Abstract

Caspase-independent cell death (CICD) has recently become a very important mechanism in lung cancer, in particular, to overcome a critical failure in apoptotic cell death that is common to disease progression and treatment failures. The pathways involved in CICD span from necroptosis, ferroptosis, mitochondrial dysfunction, and autophagy-mediated cell death. Its potential therapeutic applications have been recently highlighted. Glutathione peroxidase 4 (GPX4) inhibition-driven ferroptosis has overcome drug resistance in non-small cell lung cancer (NSCLC). In addition, necroptosis involving RIPK1 and RIPK3 causes tumor cell death and modulation of immune responses in the tumor microenvironment (TME). Mitochondrial pathways are critical for CICD through modulation of metabolic and redox homeostasis. Ferroptosis is amplified by mitochondrial reactive oxygen species (ROS) and lipid peroxidation in lung cancer cells, and mitochondrial depolarization induces oxidative stress and leads to cell death. In addition, mitochondria-mediated autophagy, or mitophagy, results in the clearance of damaged organelles under stress conditions, while this function is also linked to CICD when dysregulated. The role of cell death through autophagy regulated by ATG proteins and PI3K/AKT/mTOR pathway is dual: to suppress tumor and to sensitize cells to therapy. A promising approach to enhancing therapeutic outcomes involves targeting mechanisms of CICD, including inducing ferroptosis by SLC7A11 inhibition, modulating mitochondrial ROS generation, or combining inhibition of autophagy with chemotherapy. Here, we review the molecular underpinnings of CICD, particularly on mitochondrial pathways and their potential to transform lung cancer treatment.

## Introduction

Cellular death is an intrinsic cellular mechanism essential for preserving tissue equilibrium, removing impaired cells to combat infection and subsequent cancer, and restricting the proliferation of cancerous cells, which is key to treating and healing cancer (Loftus et al. 2022). Among the mechanisms of cell death, apoptosis and CICD hold unique functions under healthy and pathological circumstances (Galluzzi et al. 2018). Caspases, a family of cysteine proteases, are most widely studied for orchestrating apoptosis (Lam and Pozo 2000). Cellular events involved include the formation of apoptotic bodies, DNA fragmentation, chromatin condensation, and phagocyte clearing (Prokhorova et al. 2015). One hallmark of cancer is dysregulation of apoptosis, the process by which a cell’s fate is dictated even when it is not functional and cells are not kept viable with continuous delivery of oxygen and nutrients (He et al. 2024). Conversely, CICD depicts a path of cell elimination independent of the failure of apoptotic machinery (Tait and Green 2008). This mode of cellular death encompasses mechanisms like necroptosis, ferroptosis, and autophagy-dependent cellular death (Gao et al. 2022). These bypasses differ in the distinct molecular routes they share without activating the caspase (Laukens et al. 2011). CICD-related mechanisms have gained interest in cancer biology, including in lung cancer, where apoptotic resistance remains a large obstacle to effective treatment (Cao et al. 2024).

The intrinsic route is regulated by the Bcl- 2 proteins activated by cellular stress, typically DNA damage and oxidative stress (Susnow et al. 2009). When ligands attach to death receptors, the extrinsic route is started, affecting the activation of caspases (Kumar et al. 2005). However, while these pathways are important, they are often disabled in cancers by mutations in master regulators such as TP53 and Bcl- 2, which allow for unbridled proliferation (Hemann and Lowe 2006). In addition, mitochondrial dysfunction, the underlying cause of many cancers, enhances resistance to apoptosis and fuels tumor progression by modifying energy redox homeostasis and cytochrome c release (Hsu et al. 2016). These apoptotic barriers are circumvented by CICD pathways to provide alternative routes for cancer cells resistant to apoptosis to exhibit cell death (Wang et al. 2020). Necrosis is controlled by iron and is called ferroptosis and is characterized by lipid peroxidation and oxidative damage (Li et al. 2020). Ferroptosis is regulated by GPX4 and SLC7 A11 and has recently been recognized as a potential treatment target in drug-resistant cancer, especially in NSCLC (Chen et al. 2023a). Another CICD pathway, necroptosis, is created by RIPK1, RIPK3, and MLKL and can trigger inflammatory responses that promote anti tumor immunity (Galluzzi et al. 2017). Similarly, the lysosomal breakdown of cellular components and autophagy-dependent cell death play two roles in cancer, resulting in either the promotion or suppression of tumor growth depending on context (Yun et al. 2020).

CICD mechanisms are critical in overcoming therapeutic resistance in pulmonary carcinoma (Xiang et al. 2024a). In NSCLC, the predominant subtype of lung cancer, mutations in apoptotic regulators, such as TP53, allow tumors to escape conventional therapies (Canale et al. 2022). Inhibiting GPX4 or depleting glutathione triggers ferroptosis, while necroptosis activation via RIPK1 and RIPK3 cell death pathways potentiates anti-tumor immune responses (Tong et al. 2022). Additionally, mitochondrial pathways are central to CICD, as many cases of mitochondrial stress cause the release of DAMPs reactive nodes of inflammation and immunity within the TME (Kuo et al. 2024). Apoptosis and CICD are central to developing new therapies for lung cancer (Paul and Jones 2014). Targeted therapies that include Bcl- 2 inhibitors have improved apoptosis, yet the development of resistance has constrained the utility of these therapies (Lampson and Davids 2017). Complementary strategies based on CICD targeting circumvent the limitations of the apoptosis-focused strains and provide additional possible avenues in combination therapy (Hersey and Zhang 2009). Furthermore, CICD influences immune modulation and may have beneficial synergistic effects when combined with immunotherapies to augment anti-tumor effects (Varayathu et al. 2021).

This review identifies ferroptosis, necroptosis, autophagy, and mitochondrial dysfunction-related pathways during CICD in lung cancer and their implications in therapeutics. This review also aims to summarize the therapeutic strategies designed to overcome the resistance and improve the efficacy of treatment in this complicated disease by exploring the interplay of CICD and lung cancer progression.

## Role of CICD in lung cancer

One characteristic of lung carcinoma is the avoidance of apoptosis, which plays a critical role in its progression, therapeutic resistance, and poor prognosis (Fernald and Kurokawa 2013; Bhat et al. 2022). Much of the disruption of the apoptotic function of lung cancer occurs by means including mutations in the TP53 gene, overexpression of Mcl- 1 and Bcl- 2, and others (Chen et al. 2025). Through the intrinsic apoptotic pathway, a crucial tumor suppressor called TP53 raises Bax and Bak levels while lowering anti-apoptotic proteins (Wong 2011). Still, mutations in the TP53 gene decrease its capacity to trigger apoptosis, permitting carcinoma cells to escape apoptosis (Muller and Vousden 2014). Furthermore, overexpressing the Bcl- 2 proteins family in parallel inhibits MOMP, an essential step for apoptosis, exacerbating resistance to cell death (Lalier et al. 2022). This resistance cripples the effectiveness of conventional therapies, chemotherapy, and radiation, which invariably induce apoptosis, the process by which cancer cells die (Shahar and Larisch 2020). As a result, the mechanism of CICD has become a promising strategy against therapeutic resistance in lung cancer (Kang et al. 2024).

CICD provided alternative pathways for inducing cellular death in lung carcinoma cells. Unlike apoptosis, CICD includes several mechanisms, including necroptosis, ferroptosis, and autophagy-dependent cell death, independent of caspase activation (Peng et al. 2022; Wang et al. 2023a). RIPK1, RIPK3, and MLKL mediate Necroptosis, which releases Damage-associated molecular patterns (DAMPs) into the TME, causing an inflammatory reaction (Kaczmarek et al. 2013). GPX4 and SLC7 A11 govern ferroptosis, which occurs through lipid peroxidation dependent on iron and can potentially overcome the inability to respond to targeted treatments like chemotherapy (Ma et al. 2022). ATG proteins mediate autophagy-dependent cell death, a stress survival versus cell death switch (Liu et al. 2023).

CICD pathways in lung cancer are regulated, and efficacy is not only influenced but is also significantly influenced by the TME (Wang et al. 2023b). Indeed, hypoxia, a defining characteristic of the TME in solid tumors, stimulates therapeutic resistance by activating adaptive responses by hypoxia-inducible factors (HIFs) (Emami Nejad et al. 2021). These factors modulate key CICD regulators, for example, SLC7 A11 and Beclin1, to allow cancer cells to resist ferroptosis and autophagy-dependent cell death (Li et al. 2024a). The HIF-dependent increased SLC7 A11 expression increases glutathione synthesis and cystine uptake to suppress ferroptosis (Koppula et al. 2021). Although hypoxia-induced autophagy, like autophagy, helps cancer cells adjust to nutritional shortage, excessive autophagy can result in cell death dependent on autophagy (Zaarour et al. 2021). Consequently, CICD may increase lung cancer cells’ susceptibility to pathways linked to hypoxia (Chen et al. 2023b).

The mechanisms by which CICD is conducted in the TME are also modulated by metabolic stress in the TME (Czajka-Francuz et al. 2023). Metabolic phenotypes of lung cancer cells show increased reliance on aerobic glycolysis and glutaminolysis to meet their biosynthetic and energetic demands (Huang et al. 2024). These metabolic adaptations modulate CICD pathways, mainly ferroptosis and necroptosis (Fu et al. 2024). Glutaminolysis would help produce glutathione, the key antioxidant, and prevent lipid peroxidation and ferroptosis (Xu et al. 2021). Enhancing ferroptosis and suppressing tumor growth via inhibiting glutaminolysis or targeting metabolic vulnerabilities, such as cystine transport, have been demonstrated (Lei et al. 2022). In addition, autophagy committed in the setting of metabolic stress promotes intracellular recycling of nutrients, which is crucial for cancer cell survival. However, excessive activation may trigger autophagy-dependent cell death (Altman and Rathmell 2012). Redox homeostasis within the TME dictates the behavior of CICD mechanisms tightly. Within the organ system, ROS generation can worsen to either excess or deficiency, for example, in cases of mitochondrial dysfunction or ferroptosis, which tip the balance from survival to cell death (Lewerenz et al. 2018). This is exemplified by lipid ROS that, in ferroptosis, induce tumor cell death and regulate immune responses by oxidizing tumor signaling lipid and protein (Shi et al. 2022). In addition, autophagy is critical to clear damaged mitochondria to buffer oxidative stress, but its dysregulation might shift redox equilibrium and promote CICD (Schirrmacher 2020). These oxidative dynamics are shown to modulate the sensitivity of CICD cells to stress conditions and the fate of cancer cells. Directly, lung cancer’s metabolic rewiring, including enhanced glutaminolysis and lipid synthesis, alters the sensitivity to CICD (Xiong 2024). The loss of glutathione plays an important role in sustaining glutaminolysis and fuelling glutathione production, whereas glucose deprivation may lead to an autophagy-dependent cell death (Lin et al. 2012). SLC7 A11 and HIF- 1α are hypoxic conditions that modulate SLC7 A11 and HIF- 1α to confer resistance to ferroptosis (Fan et al. 2021). Intracellular metabolites such as fumarate and succinate alter redox sensitive CICD pathways through affecting intracellular ROS levels (Chen et al. 2009). Therefore, targeting metabolic dependencies may indicate CICD induction and may restrict tumor progression.

CICD pathways are also affected by the other hallmark of the TME, immune evasion (Galassi et al. 2024). Cancer cells evade immune surveillance by recruiting immunosuppressive cells, including Myeloid-derived suppressor cells (MDSCs) and Treg, and upregulating immunological check point markers like PD-L1 (Hou et al. 2020). CICD pathways, including necroptosis, can overcome immune evasions and release DAMPs, like ATP and HMGB1, to trigger dendritic cells and cytotoxic T cells to promote anti-tumor immunity (Sprooten et al. 2020). Nevertheless, necroptosis-associated inflammation may join in with promoting tumor progression via angiogenesis and metastasis (Hsu et al. 2020). Consequently, necroptosis-induced inflammation must be carefully modulated to harness its therapeutic potential (He et al. 2021). The interplay between CICD and the TME holds significant therapeutic implications for lung cancer (Katic et al. 2024). Immune checkpoint inhibitors and ferroptosis inducers have been shown to work in concert in preclinical models to enhance anti-tumor immunity and cancer cell death (Cai et al. 2023). As such, modulation of autophagy or necroptosis has been demonstrated to make lung carcinoma cells resistant to chemotherapy and radiotherapy (Roy et al. 2022). Necroptosis-mediated RIPK3 activation of dendritic cells can increase maturation and improve immune responses (Zhao et al. 2020). In addition, exploiting metabolic vulnerabilities present in the TME by targeting glutaminolysis or cystine transport may further promote ferroptosis while retaining little ability to resist therapy (Zheng and Conrad 2020). CICD mechanisms such as necroptosis and ferroptosis play dual roles in immune modulation within the TME. Release of DAMPs such as HMGB1 and ATP via necroptosis activates antigen-presenting cells and CD8 + T cells, promoting the elevation of anti-tumor response (Garg and Agostinis 2017). Yet, persistent necroptosis-induced inflammation may attract MDSC and Tregs for immunoevasion by engendering an immunosuppressive niche (Awadasseid et al. 2021). Furthermore, ferroptosis-induced lipid peroxidation produces oxidized phospholipids that could diminish dendritic cell maturation, thereby enhancing immune escape. This dynamic interplay shapes the immunogenicity of the tumor and its immunotherapies (Xu et al. 2021).

## Mechanisms of caspase-independent cell death

### Mitochondrial pathways

**Fig. 1:**
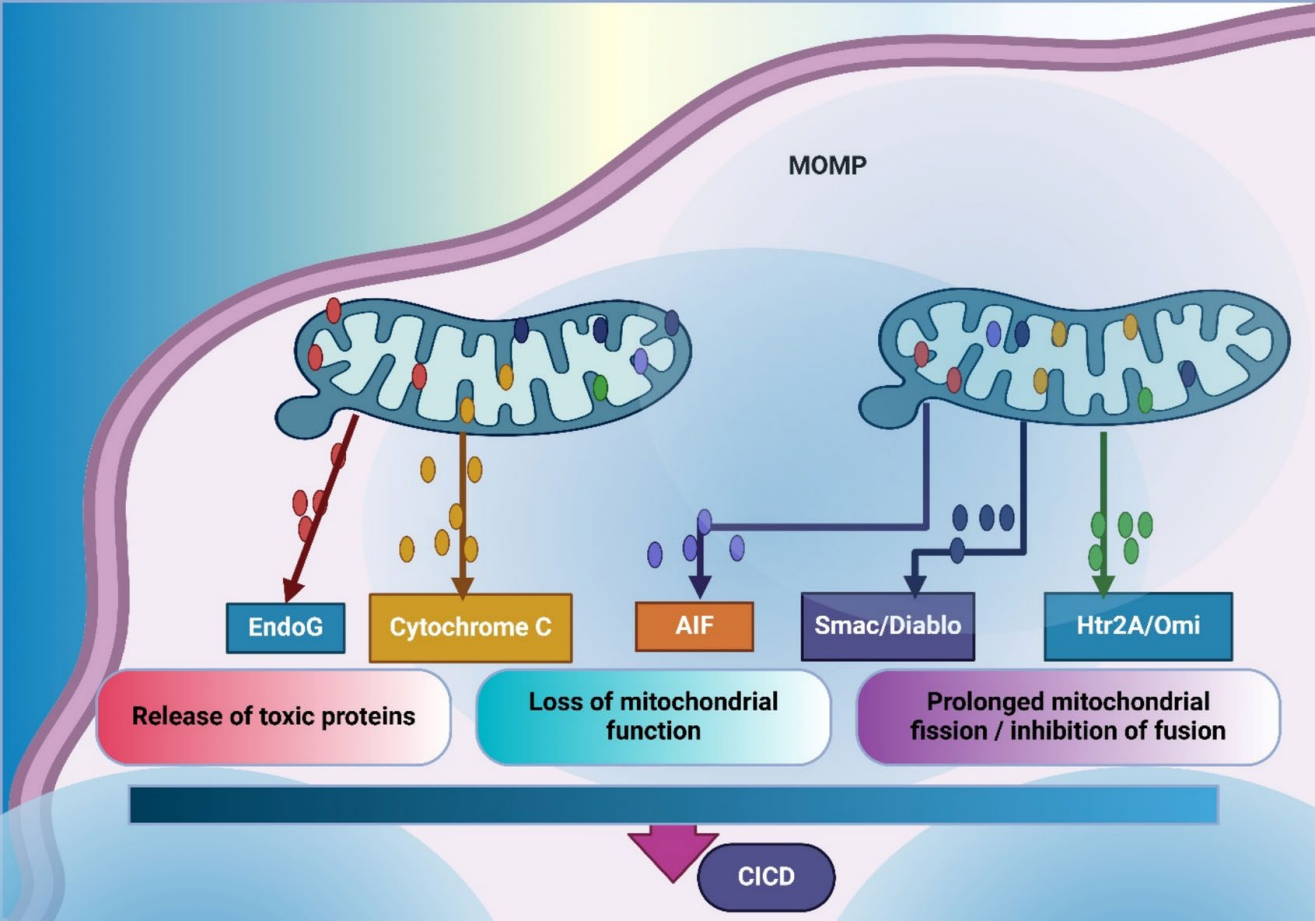
Illustrates the mitochondrial outer membrane permeabilization (MOMP) process and its role in cell death through the release of various mitochondrial proteins. Upon MOMP induction, toxic proteins such as EndoG and cytochrome C are released, leading to apoptotic or necrotic pathways. Cytochrome C release is associated with the loss of mitochondrial function, while apoptosis-inducing factor (AIF) contributes to mitochondrial dysfunction. Smac/Diablo and Htr2 A/Omi promote prolonged mitochondrial fission and inhibit fusion, further exacerbating mitochondrial instability

CICD, an apoptosis-like mode of cell death, goes through a caspase-independent route engaged by mitochondrial dysfunction (Pradelli et al. 2010). This process includes Mitochondrial outer membrane permeabilization (MOMP), the release of apoptosis-inducing molecules such as EndoG and AIF, and the production of ROS, as shown in Fig. 1 (Yapryntseva et al. 2024). CICD occurs in caspase-dependent apoptosis when classical apoptotic signaling pathways are inhibited, as occurs, for example, when viral infections or caspase inhibitors are used (May and Madge 2007; Bhat et al. 2024). CICD is critical in cancer biology and, most notably, in lung cancer progression and therapy by exploiting alternative death pathways in apoptosis-resistant cells (Gong et al. 2019). The MOMP is a critical and central step in developing CICD, initiating the discharge of AIF and EndoGo from mitochondria (Wang and Youle 2009). Following the release, AIF moves into the nucleus, where it causes chromatin condensation and extensive DNA breakage, followed by EndoG amplifying DNA degradation (Sevrioukova 2011). Oxidative damage caused by hydrogen peroxide is induced when ROS saturate, and they act as a signaling molecule at lower levels (Jomova et al. 2023). MOMP and pro-death signaling cascades are initiated by ROS (Wu and Bratton 2013). Yang et al. demonstrated that curcumin induces ROS-mediated apoptosis in SCLC by disrupting mitochondrial membrane potential, upregulating Bax, and decreasing Bcl-xL and Bcl- 2 (Yang et al. 2012). Similarly, bortezomib, a proteasome inhibitor, enhances ROS production, destabilizes mitochondrial membranes, and induces cytochrome c release in lung cancer cells (Ling et al. 2003). Other regulators of CICD include key signaling molecules such as p38MAPK (Koul et al. 2013). Song et al. showed that Baohuoside I induces apoptosis in NSCLC by activating the ROS/p38MAPK pathway, disrupting mitochondrial membrane potential, and triggering caspase activation (Song et al. 2012). Additionally, Li et al. revealed that swainsonine triggers mitochondrial apoptosis in A549 cells by promoting PARP cleavage and activating pro-apoptotic signaling, further linking mitochondrial dysfunction with DNA repair processes (Li et al. 2012).

Mitochondrial metabolic reprogramming plays a significant role in CICD (Fan et al. 2024). Dysregulated genes involved in energy metabolism and OXPHOS contribute to cancer progression and therapy resistance (Tufail et al. 2024). Ye et al. analyzed MMRGs in lung carcinoma, identifying 43 differentially expressed genes, including GAPDHS, linked to a bad prognosis (Ye et al. 2021). Another critical regulator is SLC25 A4, an ATP/ADP exchanger (Ruprecht and Kunji 2020). Hertweck et al. demonstrated that altered SLC25 A4 expression in NSCLC is linked to tumor growth and survival, highlighting its therapeutic potential (Hertweck et al. 2023). Bioenergetic phenotypes further influence CICD (Hill et al. 2019). Han et al. revealed that OXPHOS phenotypes determine metabolic dependencies and therapeutic outcomes in NSCLC, suggesting a direct link between mitochondrial bioenergetics and cancer progression (Han et al. 2023). Additionally, Zhang et al. showed that mitochondria-targeted honokiol (Mito-HNK) combined with lonidamine (Mito-LND) disrupts oxidative phosphorylation, leading to an 83% reduction in tumor burden in murine models (Zhang et al. 2022a).

It has been demonstrated that several synthetic and natural substances can cause CICD in lung carcinoma by focusing on mitochondrial pathways. *Vitexin*, a plant-derived flavonoid, inhibits PI3 K/Akt/mTOR signaling, promotes apoptosis, and reduces mitochondrial membrane potential in NSCLC (Liu et al. 2019). Piperine, another natural compound, selectively induces mitochondrial apoptosis in A549 cells by activating Bax and caspases while sparing normal fibroblasts, demonstrating its potential as a selective chemotherapeutic agent (Lin et al. 2014). Similarly, Dioscin, a steroidal saponin, triggers mitochondrial structural changes and S-phase arrest, further emphasizing the importance of mitochondrial signaling in lung cancer (Wei et al. 2013). In addition to natural compounds, Zhao et al. highlighted the efficacy of *dehydrobruceine B* (DHB) in inducing mitochondrial dysfunction and apoptosis in NSCLC cells via caspase- 9 activation (Zhao et al. 2016a). Kim et al. showed that *Trichostatin A* (TSA) induces apoptosis in NSCLC cells by simultaneously triggering extrinsic and intrinsic cascade, underscoring its potential for targeting multiple apoptotic mechanisms (Kim et al. 2006).

Mitochondrial adaptation is a key contributor to therapy resistance in lung cancer. Nicotine, for instance, impairs chemotherapy-induced apoptosis in A549 cells by stabilizing mitochondrial membrane potential and activating Akt-mediated anti-apoptotic proteins (Zhang et al. 2009). Zhou et al. developed mitochondrial-targeting paclitaxel liposomes that selectively accumulate in mitochondria, overcoming resistance in cisplatin-resistant A549 cells by promoting Bax activation and suppressing Bcl- 2 (Zhou et al. 2013). Emerging therapeutic strategies also target regulators like Bcl-XL, a prominent anti-apoptotic protein (Valentini et al. 2022). Doi et al. showed that FR901228 downregulates Bcl-XL and enhances caspase activation, thus becoming a viable choice for overcoming therapy resistance in SCLC (Doi et al. 2004).

Targeting mitochondrial pathways offers significant therapeutic potential but poses challenges, including therapy resistance and off-target toxicity (Li et al. 2024b). Combining ROS modulators with mitochondrial-targeted agents may improve efficacy (Okon and Zou 2015). For example, Liu et al. demonstrated that HSP-III induces the generation of ROS and mitochondrial apoptosis in NSCLC cells, highlighting the promise of ROS-modulating therapies (Liu et al. 2017). Furthermore, Han et al. identified the role of dysfunctional mitochondria in EMT, linking it to metastasis and suggesting therapeutic interventions to reverse these phenotypes (Han et al. 2018). Gao et al. additionally disclosed that the downregulation of GSDMD suppresses tumor growth by promoting mitochondrial apoptosis and inhibiting EGFR/Akt signaling, identifying GSDMD as a promising therapeutic target (Gao et al. 2018) (Fig. 1).

### Necroptosis

Necroptosis differs from apoptosis because it is caspase-independent (Bertheloot et al. 2021). RIPK3 and RIPK1 mediate the formation of a necrosome complex, which primarily mediates necroptosis (Morgan and Kim 2022). RIPK3 phosphorylates MLKL, which causes the protein to oligomerize and translocate to the plasma membrane, disrupting the integrity of the membrane (Wang et al. 2014). This mechanism causes inflammation by inducing cell lysis and releasing intracellular substances (Rock and Kono 2008). Through dysregulation, necroptosis in cancer can lead to tumor growth, inflammation, and immunological suppression, in addition to serving as a fallback mechanism when apoptosis is suppressed (Della Torre et al. 2021; Thapa et al. 2023). However, it contains therapeutic potential in exploiting its pathways to overcome apoptosis resistance and augment immune response in cancer therapies (Baig et al. 2016). Necroptosis initiation and execution depend on RIPK1 and RIPK3 (Grootjans et al. 2017). Park et al. demonstrated significantly reduced MLKL, RIPK3, and RIPK1 levels in NSCLC cells, correlating with poor prognosis and early recurrence, especially in adenocarcinoma (Park et al. 2020). Wang et al. highlighted that RIP3 promoter methylation limits necroptosis, reducing chemotherapy efficacy. Restoring RIP3 expression sensitized NSCLC cells to chemotherapeutics such as cisplatin and etoposide (Wang et al. 2020a). MLKL, the terminal effector of necroptosis, plays a pivotal role in disrupting membrane integrity (Zhan et al. 2021). Jing et al. revealed phosphatidylinositol transfer protein alpha (PITPα) promotes membrane translocation and MLKL oligomerization in cisplatin-induced necroptosis (Jing et al. 2018).

Necroptosis significantly impacts the TIME (Zhao et al. 2022). Zhao et al. identified necroptosis-related phenotypes in LUAD, developing a NecroScore that stratifies patients based on survival, immune response, and therapy sensitivity (Zhao et al. 2022). Duangthim et al. observed that high RIP3 expression correlates with better prognosis but creates an immunosuppressive TIME, marked by reduced CD8 + T cells and increased macrophages (Duangthim et al. 2024). Long et al. identified NO.0449–0145, an APE1 inhibitor, as a necroptosis inducer, apoptosis, and pyroptosis in NSCLC cells. This substance also overcame cisplatin and erlotinib resistance (Long et al. 2021). Zhao et al. explored isobavachalcone derivatives, finding that compound 16 induces necroptosis alongside apoptosis through ROS accumulation and mitochondrial dysfunction (Chen et al. 2024a). Tan et al. reported that ID1 overexpression enhances gefitinib sensitivity by promoting RIP1/RIP3/MLKL-dependent necroptosis (Tan et al. 2020).

Necroptosis-related genes and miRNAs emerge as prognostic tools in NSCLC (Hong et al. 2022a). Lim et al. identified low expression of RIPK3 and PELI1 as indicators of poor survival in squamous cell carcinoma (Lim et al. 2021). Dai and Fu developed a six-gene necroptosis-related signature, including RIPK3 and MLKL, stratifying patients based on prognosis and immune activity (Dai and Fu 2022). Hong et al. constructed a miRNA-based nomogram incorporating necroptosis-related risk scores for LUAD, improving survival predictions (Hong et al. 2022b). Zhu et al. analyzed necroptosis-associated genes in GEO and TCGA datasets, linking necroptosis signatures to chemotherapy sensitivity and prognosis (Zhu et al. 2022).

Several substances have been demonstrated to cause necroptosis in NSCLC (Chen et al. 2016). Shikonin triggers RIP1-dependent necroptosis, and Kim et al. demonstrated enhanced efficacy when combined with autophagy inhibitors like ATG5 siRNA, 3-MA, or bafilomycin A (Kim et al. 2017). Acetylshikonin induces necroptosis through phosphorylation of MLKL, RIPK3, and RIPK1, accompanied by mitochondrial dysfunction and ATP depletion (Lin et al. 2023). *Deoxypodophyllotoxin* (DPT) inhibits NSCLC cell proliferation and induces necroptosis via mitochondrial dysfunction and ROS generation, proving effective in drug-sensitive and resistant cells (Wu et al. 2013). Similarly, citronellol promotes necroptosis through TNF-α and biphasic production of ROS, inhibiting tumor growth in xenograft models (YU et al. 2019). LGH00168, a novel CHOP activator, induces RIP1-dependent necroptosis via ROS-mediated NF-κB inhibition and ER stress, significantly reducing tumor growth in vivo (Ma et al. 2016). HS- 173 selectively activates RIP3 and MLKL, inducing necroptosis in RIP3-expressing NSCLC cells and suppressing tumor growth in mouse models (Park et al. 2019). Silibinin (SiL) also triggers apoptosis and necroptosis via RIPK1, RIPK3, and MLKL activation, significantly reducing NSCLC tumor growth in vivo (Guoqing et al. 2024).

The dual roles of necroptosis in cancer offer both challenges and opportunities. Novel strategies combining inducers of necroptosis, like MAM (2-methoxy- 6-acetyl- 7-methyljuglone), with immunotherapy may enhance anti-tumor responses (Sun et al. 2019). NecroLncSig models, as proposed by Lin et al., represent a promising approach for integrating necroptosis biomarkers into personalized treatment strategies (Lin et al. 2024). Lastly, dexmedetomidine-induced PARP1 activation can potentially target necroptosis pathways for NSCLC treatment (Liu et al. 2024).

### Autophagic cell death (ACD)

ACD is a CICD in which cellular death culminates through excessive autophagy (Kroemer and Levine 2008). ACD differs from apoptosis, which is mainly caspase-dependent, in that it is mediated by the accumulation of autophagosomes and autolysosomes elicited by cellular stressors, including nutrient deprivation, hypoxia, or therapeutic agents (Mariño et al. 2014). ACD can serve as a tumor suppressor, which clears damaged cells, but dysregulated autophagy can also convey tumor progression or therapy resistance (Patra et al. 2022). Pivotal roles of regulators, including Beclin- 1, ATG proteins, and the PI3 K/AKT/mTOR cascade, in regulating ACD are indicated, offering therapeutic possibilities, especially in cancers resistant to caspase-related therapies (Pang et al. 2021; Thapa 2024). Autophagy is an example of autophagy’s dual nature because of the critical function ATG5 (Pua et al. 2007). Rao et al. showed that ATG5 deletion in a KRasG12D-driven lung cancer model impaired tumor progression by inducing oxidative stress and mitochondrial dysfunction. However, loss of ATG5 accelerated early tumor onset through regulatory T-cell involvement, highlighting the complex role of autophagy in tumor development (Rao et al. 2014). Similarly, p62/SQSTM1 plays a multifaceted role in cancer (Tang et al. 2021). Liu et al. reported that curcumin-induced autophagy in NSCLC cells reduced p62 levels, downregulated the PI3 K/AKT/mTOR cascade, and enhanced apoptosis (Liu et al. 2018).

The mTOR signaling cascade is a central regulator of autophagy, providing therapeutic targets for intervention (Zou et al. 2020). Wang et al. reported that ursolic acid (UA) hindered the mTOR pathway, inducing apoptosis and autophagy in NSCLC cells. Autophagy antagonist co-treatment increased UA’s anticancer properties (Wang et al. 2020b). Peng et al. found that alpha-lipoic acid suppressed tumor growth in NSCLC by activating mTOR-mediated inhibition of autophagy (Peng et al. 2020). Furthermore, Kim et al. reported that RAD001 (everolimus), an mTOR inhibitor, enhanced radiosensitivity by promoting autophagy in NSCLC models (Kim et al. 2008).

**Fig. 2:**
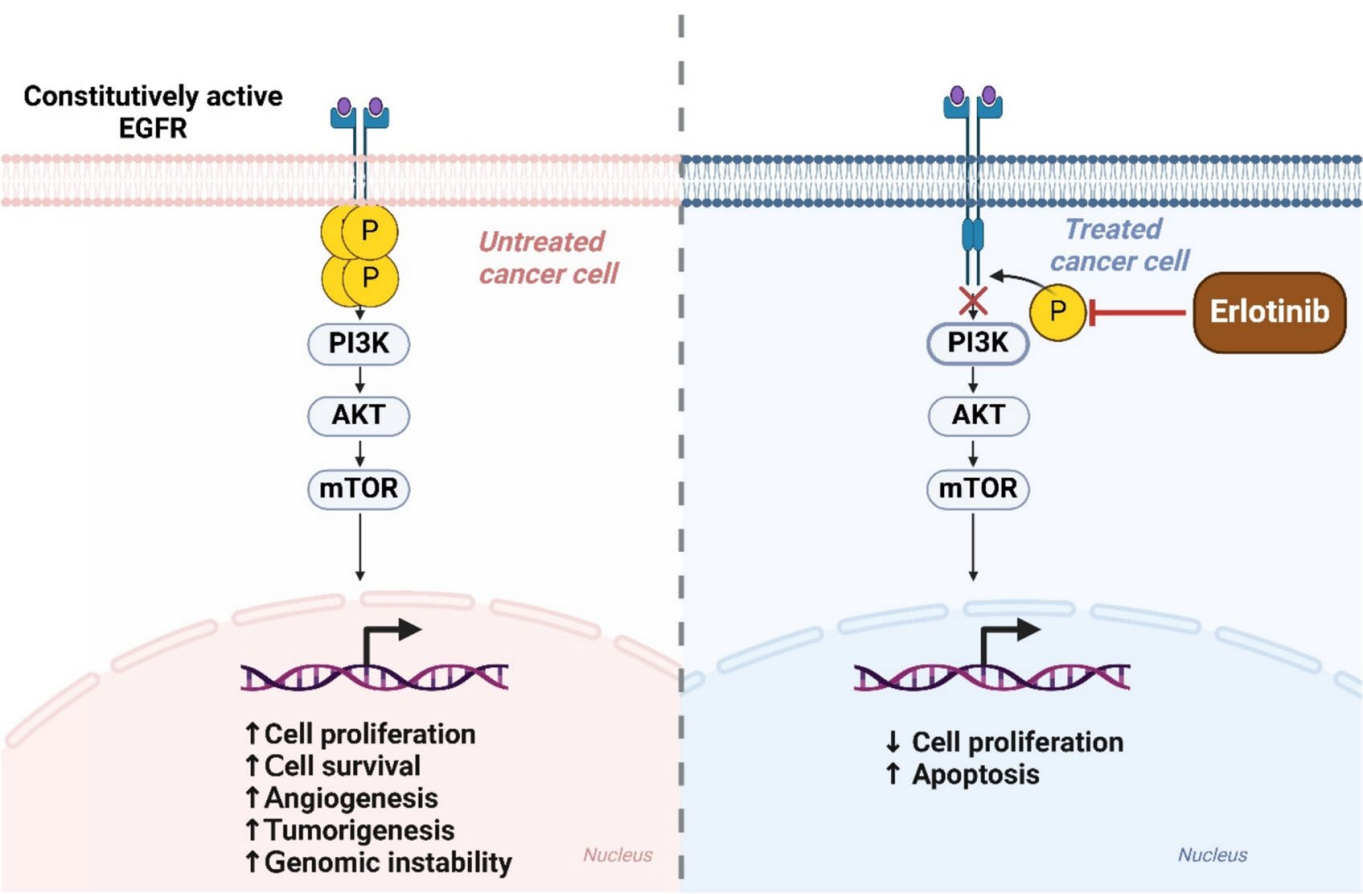
Illustrates Erlotinib’s mechanism of action in inhibiting EGFR signaling in cancer cells. An untreated cancer cell with constitutively active EGFR undergoes phosphorylation, activating the PI3 K/AKT/mTOR pathway and increasing cell proliferation, survival, angiogenesis, tumorigenesis, and genomic instability. Erlotinib treatment inhibits EGFR phosphorylation, blocking downstream signaling. This suppression of the PI3 K/AKT/mTOR pathway results in decreased cell proliferation and increased apoptosis, demonstrating Erlotinib’s role in cancer therapy by targeting EGFR-dependent survival mechanisms

Autophagy-related ROS production links autophagy to cell death mechanisms (Filomeni et al. 2015). Kaminskyy et al. reported that autophagy inhibition increased ROS levels, amplifying cisplatin-induced apoptosis in NSCLC (Kaminskyy et al. 2012). Similarly, Xie et al. demonstrated that acid-induced autophagy, facilitated by ROS and ER stress, promoted cell survival in NSCLC, while autophagy inhibition enhanced apoptosis (Xie et al. 2015). miRNAs that regulate autophagy have been identified as potential therapeutic targets (Bhat et al. 2023). Rezaei et al. highlighted miRNAs that modulate autophagy and influence NSCLC progression, offering novel avenues for therapy (Rezaei et al. 2020). Zhao et al. reported that gefitinib-induced autophagy contributed to resistance in glucocorticoid-resistant NSCLC cells (Zhao et al. 2016b). Zou et al. demonstrated that the autophagy inhibitor chloroquine enhanced the efficacy of erlotinib in NSCLC with wild-type EGFR by inhibiting cytoprotective autophagy, as shown in Fig. 2 (Zou et al. 2013). Additionally, Wang et al. explored how mTOR and PI3 K/AKT regulate autophagy in NSCLC, highlighting their therapeutic potential (Wang et al. 2022a).

Therapeutic targeting of autophagy has shown potential for overcoming drug resistance (Chang and Zou 2020). EGFR-TKIs such as gefitinib and erlotinib activate cytoprotective autophagy, limiting their efficacy (Fung et al. 2012). Han et al. revealed that inhibiting autophagy with chloroquine or ATG5/ATG7 siRNAs significantly enhanced EGFR-TKI-induced cytotoxicity in NSCLC cells (Han et al. 2011). Similarly, Tang et al. demonstrated that combining dacomitinib with cepharanthine, an autophagic inhibitor, enhanced tumor suppression in NSCLC (Tang et al. 2018). Furthermore, TUDC increased cisplatin-induced apoptosis by mitigating autophagy and ER stress, as reported by Shi et al. (Shi et al. 2016). Hypoxia-induced autophagy is a significant therapy resistance mechanism (Hill et al. 2023). Wu et al. reported hypoxia-enhanced autophagy through HIF- 1α and HIF- 2α, promoting cisplatin resistance in NSCLC. Inhibiting autophagy with 3-MA or ATG5 siRNA restored drug sensitivity (Wu et al. 2015). Similarly, Lee et al. demonstrated that hypoxia-induced autophagy contributed to cisplatin resistance, which was reversed by LC3B knockdown (Lee et al. 2015). Radiation-induced autophagy has also been linked to resistance mechanisms (Classen et al. 2019). Karagounis et al. showed that silencing LC3B and LAMP2a autophagy inhibition enhanced both radiosensitivity and chemosensitivity in NSCLC cells (Karagounis et al. 2016). Wang et al. demonstrated that cardiac glycosides promoted autophagy through AMPK and ERK1/2 activation, inducing tumor suppression in NSCLC cells (Wang et al. 2012). SQSTM1/p62 and ATG7-dependent pathways have been explored for their roles in therapeutic resistance (Ma et al. 2019). Zhang et al. revealed that w09, an investigational autophagy inducer, caused autophagy that was both ATG7-independent and dependent, which encouraged apoptosis in NSCLC (Zhang et al. 2021a; Lou et al. 2024).

Several novel compounds have been identified as autophagy inhibitors (Konstantinidis et al. 2019). Zhang et al. identified CA- 5f, a curcumin-derived compound, as a late-stage autophagy inhibitor that caused NSCLC cells to undergo selective apoptosis by producing ROS in the mitochondria (Zhang et al. 2019). Chen et al. showed that juglanin, derived from *Polygonum aviculare*, induced apoptosis and autophagy via TRAIL/DR activation and ROS modulation, suppressing NSCLC tumor progression (Chen et al. 2017). In NSCLC, autophagic cell death is a multifaceted process that serves as a mediator of resistance and a tumor suppressor. Targeting autophagy through inhibitors, modulation of mTOR and ROS pathways, or miRNA modulation can potentially improve treatment results and overcome medication resistance. Continued research into the molecular underpinnings of autophagy will provide new opportunities for therapeutic innovation in NSCLC (Fig. 2).

### Ferroptosis

Iron-dependent lipid peroxidation drives ferroptosis, a controlled form of CICD (Tang et al. 2021a). Ferroptosis differs from apoptosis because it involves ROS accumulation and oxidative membrane damage of polyunsaturated fatty acids (Stockwell 2022). GSH depletion and GPX4 inhibition cause enhancement of oxidative stress, which are key drivers (Li et al. 2022). It is regulated intricately by pathways in which iron homeostasis, lipid metabolism, and antioxidant defence pathways perform a crucial part. While preventing the formation of tumors by eliminating malignant cells, cancer cells develop resistance to ferroptosis, which is an additional therapeutic challenge (Zhou et al. 2024; Sun et al. 2023). The cystine/glutamate antiporter SLC7 A11, a crucial ferroptosis regulator, preserves redox equilibrium and shields cells from oxidative damage (Lee and Roh 2022). Ferroptosis is regulated by the RNA-binding protein RBMS1, according to Zhang et al., which enhances SLC7 A11 translation and promotes ferroptosis resistance in lung cancer—targeting RBMS1-sensitized cells to radiotherapy, highlighting it as a therapeutic target (Zhang et al. 2021). Similarly, It has been demonstrated that SOX2 protects cancer stem-like cells against ferroptosis by upregulating SLC7 A11. Oxidation at SOX2’s Cys265 site disrupted its activity, sensitizing cells to lipid peroxidation and ferroptosis (Wang et al. 2021).

**Fig. 3:**
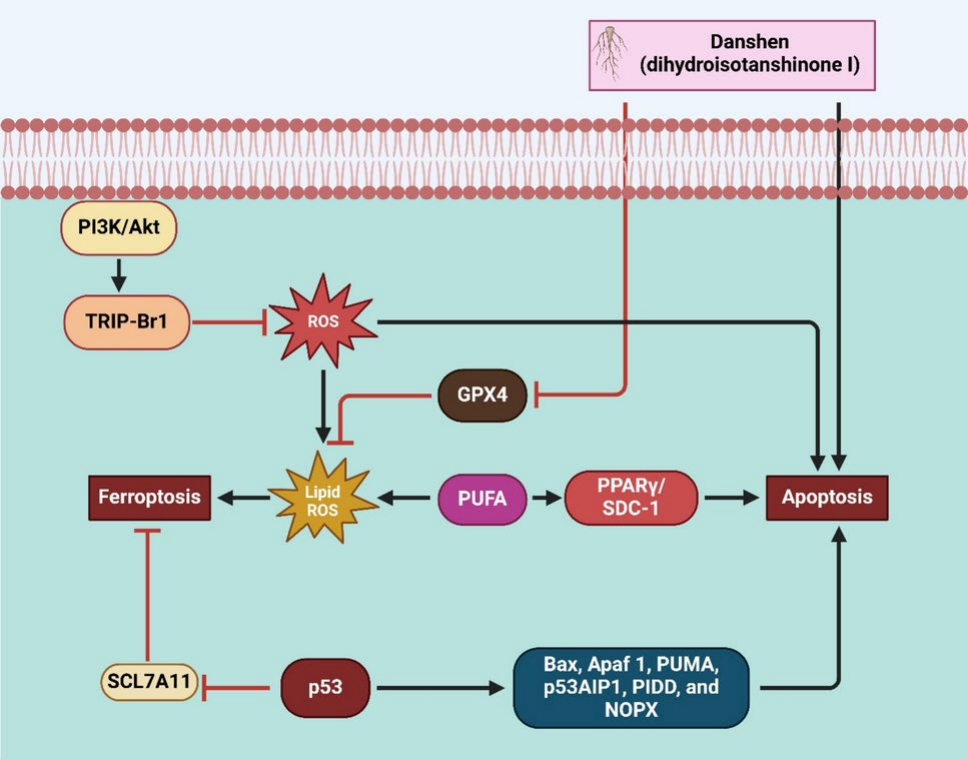
Illustrates the crosstalk between ferroptosis and apoptosis, highlighting the role of Danshen (dihydroisotanshinone I) in modulating these cell death pathways. PI3 K/Akt signaling activates TRIP-Br1, which inhibits ROS accumulation. Excess ROS triggers ferroptosis through lipid ROS production, while GPX4 inhibits ROS-induced lipid peroxidation. PUFA enhances ferroptosis via PPARγ/SDC- 1, which also promotes apoptosis. p53 induces ferroptosis by inhibiting SCL7 A11 and promotes apoptosis by upregulating Bax, Apaf- 1, and PUMA. Danshen directly enhances apoptosis and suppresses GPX4, amplifying oxidative stress to drive cell death

GPX4 inhibition is central to ferroptosis induction, making it a critical therapeutic target (Pan et al. 2022). Wu et al. demonstrated that dihydroisotanshinone I (Danshen) causes ferroptosis and inhibits GPX4 expression in lung carcinoma models, as shown in Fig. 3 (Wu et al. 2021). Similarly, bufotalin (BT) targets GPX4, inducing ferroptosis by increasing Fe^2^⁺ levels and lipid ROS in pulmonary carcinoma cells (Zhang et al. 2022b). Kim et al. further emphasized how susceptible GPX4-overexpressing cells are to RSL3 and other ferroptosis stimulants, revealing a therapeutic strategy for NSCLC (Kim et al. 2023). Ferroptosis modulation is another important function of the NRF2 signaling cascade (Dodson et al. 2019; Lin et al. 2016). Hsieh et al. demonstrated that ZVI-NPs degrade NRF2 and promote ferroptosis while reprogramming the TME to strengthen immunity against tumors (Hsieh et al. 2021). Conversely, KEAP1 mutations lead to constitutive NRF2 activation, driving ferroptosis resistance (Scalera et al. 2022). The CoQ-FSP1 axis was found by Lei et al. to be a crucial modulator of resistance in lung tumors with KEAP1 mutations, with its inhibition sensitizing tumors to radiation (Koppula et al. 2022).

Targeting lipid metabolism also enhances ferroptosis (Liang et al. 2022). According to Tang et al., curcumin causes ferroptosis in NSCLC cells by boosting lipid peroxidation and controlling GPX4 and ACSL4 via autophagy activation (Tang et al. 2021b). Wang et al. revealed that KRAS-mutant lung cancers are dependent on lipid remodeling, with FASN inhibition inducing ferroptosis (Bartolacci et al. 2022). LncRNAs also perform an important function in ferroptosis regulation. LINC00336 suppresses ferroptosis by working as a ceRNA, stabilizing cystathionine-β-synthase expression via miRNA sponging (Wang et al. 2019). Similarly, NEAT1 modulates ACSL4 expression, affecting ferroptosis sensitivity in NSCLC (Wu and Liu 2021; Nie et al. 2020). Zhang et al. revealed that miR- 27a- 3p promotes NSCLC progression by regulating ferroptosis through SLC7 A11. Overexpression of miR- 27a- 3p suppressed ferroptosis, while its inhibition increased lipid ROS and cell death, identifying the miR- 27a- 3p/SLC7 A11 axis as a possible target for treatment (Lu et al. 2021). Bartolacci et al. confirmed the role of lipid remodeling in KRAS-mutant lung cancers, where blocking the Lands cycle-induced ferroptosis offers a therapeutic vulnerability (Bartolacci et al. 2022). By connecting ferroptosis regulation to patient survival outcomes, Liu et al. discovered a five-gene profile associated with ferroptosis as a predictive tool for NSCLC (Liu et al. 2021).

Ferroptosis also intersects with immune modulation (Tang et al. 2024). Jo et al. demonstrated that plasma-activated medium (PAM) induces ferroptosis by depleting ferroptosis suppressor protein 1 (FSP1), enhancing tumor cell death and immune response (Jo et al. 2022). Huang et al. further revealed that AKR1 C1 correlates with ferroptosis suppression and reduced immune cell infiltration, highlighting its capacity as an NSCLC prognostic indicator (Huang et al. 2021).

Emerging compounds such as erianin and curcumenol also induce ferroptosis. Erianin has the potential to be a natural medicinal agent since it causes ferroptosis in lung cancer cells through Ca2 +/calmodulin signaling (Chen et al. 2020; Jiang et al. 2024). Likewise, curcumenol reduces tumor development in vivo by inducing ferroptosis through modulation of the lncRNA H19/miR- 19b- 3p/FTH1 axis (Zhang et al. 2022c). Additional therapeutic strategies have emerged for enhancing ferroptosis induction (Chen et al. 2024b). Yuan et al. identified YTHDC1 as a tumor suppressor that modulates ferroptosis by regulating FSP1 mRNA stability, suggesting it as a novel therapeutic target for NSCLC (Yuan et al. 2023). Tang et al. demonstrated that USP35 stabilizes ferroportin and regulates ferroptosis, inhibiting lung cancer cells from chemotherapy (Tang et al. 2021c; Li et al. 2024c). TRIM6 was also identified as a suppressor of ferroptosis through SLC1 A5 targeting, highlighting its role in chemoresistance (Zhang et al. 2023).

Innovative delivery systems have also been developed to enhance ferroptosis induction (Xiang et al. 2024b). *Dihydroartemisinin* (DHA) and calcium phosphate were co-loaded into an inhalable liposome by Wang et al., which causes ferroptosis via a cyclic Ca2 + -burst-ER stress-ferroptosis mechanism (Fu et al. 2023; Zhao 2025). Feng et al. further advanced nebulized nanocatalytic therapy using iron nanoparticles to target cancer stem cells, enhancing their sensitivity to ferroptosis (Feng et al. 2023). Zhang et al. demonstrated that artemisinin derivatives like artesunate and dihydroartemisinin target ferroptosis by modulating xCT and TFRC, offering effective strategies against NSCLC (Zhang et al. 2021b) (Fig. 3; Table 1).

**Table 1:** This table summarizes studies exploring therapeutic compounds targeting mitochondrial pathways, necroptosis, autophagy, and ferroptosis in lung cancer

Study type	Name of the compound	Outcome	Mechanism	CICD	References
Mitochondrial pathways	Curcumin	Increased Bax, reduced Bcl- 2	ROS-mediated mitochondrial effects	ROS-based strategies	Yang et al. 2012)
	Baohuoside I	Bax/Bcl- 2 ratio increased	ROS/JNK/p38MAPK pathway	Antioxidant development	Song et al. 2012)
	Vitexin	Reduced viability, increased apoptosis	PI3 K/Akt/mTOR signaling inhibition	Therapy potential	Liu et al. 2019)
	Piperine	Activated caspase- 9 and caspase- 3	p53-dependent mitochondrial pathway	Selective tumor targeting	Lin et al. 2014)
	Honokiol and Lonidamine	83% tumor reduction observed	Mitochondrial complex inhibition	Chemoprevention strategy	Zhang et al. 2022a)
	Trichostatin A (TSA)	Dual pathway apoptosis was observed	Mitochondrial dysfunction in NSCLC	Therapeutic potential	Kim et al. 2006)
	Cold atmospheric plasma (CAP)	Apoptosis via cytochrome c release	Mitochondrial apoptosis modulation	Novel therapeutic strategy	Wang et al. 2022b)
	Dehydrobruceine B (DHB)	S-phase arrest, apoptosis induced	Mitochondrial intrinsic pathway	Therapeutic agent potential	Zhao et al. 2016a)
	Dioscin	S-phase arrest, DNA damage induced	Mitochondrial apoptosis activation	Anticancer agent potential	Wei et al. 2013)
Necroptosis	Shikonin	RIP1-mediated death enhanced	Autophagy-necroptosis synergy	Combination strategies	Kim et al. 2017)
	NO.0449–0145	Induced necroptosis and apoptosis	APE1 inhibition mechanism	Overcomes drug resistance	Long et al. 2021)
	Citronellol	Increased TNF-α, RIP1, and RIP3	TNF-α pathway activation	Necroptosis-based therapy	YU et al. 2019)
	Acetylshikonin	Promoted RIPK1/RIPK3/MLKL necroptosis	ROS and mitochondrial stress	Necroptosis-targeted therapy	Lin et al. 2023)
	LGH00168	ER stress and RIP1 necroptosis	ROS-mediated necroptosis	Necroptosis-based strategy	Ma et al. 2016)
	Silibinin	Enhanced necroptosis, mitochondrial stress	RIPK1/RIPK3 pathway activation	Dual pathway induction	Zhang et al. 2024)
Autophagic cell death	EGFR-TKIs	Reduced drug effectiveness	PI3 K/Akt/mTOR pathway effects	TKI therapy improvements	Han et al. 2011)
	Cisplatin	Increased apoptosis with inhibitors	Autophagy-apoptosis synergy	Cisplatin resistance	Shi et al. 2016)
	Piperlongumine	Enhanced apoptosis with inhibitors	PI3 K/Akt/mTOR pathway inhibition	Chemoresistant cell targeting	Kim et al. 2008)
	CA- 5f	Selectively inhibited autophagy	Autophagosome-lysosome fusion inhibition	NSCLC treatment potential	Zhang et al. 2019)
	Ursalic acid (UA)	Induced autophagy and apoptosis	mTOR pathway inhibition	Therapeutic combination	Wang et al. 2020b)
	Cepharanthine	Enhanced autophagy-apoptosis combination	Autophagy inhibition synergy	Combined therapeutic strategy	Tang et al. 2018)
Ferroptosis	Curcumin	Increased lipid peroxidation	Autophagy-ferroptosis activation	Synergistic apoptosis	Tang et al. 2021b)
	Dihydroisotanshinone I	Apoptosis and ferroptosis induced	Apoptosis-ferroptosis effects	Dual-action therapy	Wu et al. 2021)
	Artesunate and dihydroartemisinin	Downregulated xCT, increased ROS	Ferroptosis and apoptosis synergy	Drug-resistance targeting	Zhang et al. 2021b)
	Erianin	Suppressed cell growth and migration	Ca^2^⁺/CaM-mediated ferroptosis	Ferroptosis induction	Chen et al. 2020)
	Bufotalin	Induced ferroptosis via GPX4	GPX4 ubiquitination mechanism	Ferroptosis enhancement	Zhang et al. 2022b)
	Nortriptyline hydrochloride	Reduced RBMS1 promoted ferroptosis	SLC7 A11 ferroptosis evasion	Ferroptosis-based therapy	Zhang et al. 2021)

## Challenges and future directions

Translation of CICD therapies into clinical practice remains met with several challenges, which holds immense promise for lung cancer therapy targeting CICD pathways. CICD pathways such as ferroptosis, necroptosis, and autophagy-dependent cell death are subject to complex and context-dependent complex and context-dependent regulation (Zhou et al. 2020; Dong and Jiang 2024). These processes have been demonstrated to exhibit dual characteristics in cancer biology, either promoting or suppressing tumor development based on variables such as the TME, genetic changes, and metabolic context (Sever and Brugge 2015; Zhang 2024). In addition to triggering immunological responses by generating DAMPs, necroptosis also creates an environment conducive to inflammation, advancing tumor growth and metastasis. Autophagy, traditionally viewed as a cancer cell survival mechanism during stress under metabolic or therapeutic conditions, also complicates the exploitation of autophagy as a therapeutic target. Its excessive activation also provides a selective advantage for cancer cell survival (Yang et al. 2011; Pu et al. 2024). The major challenge is to balance activating CICD pathways without inducing harmful responses, such as inflammation, immune evasion, and therapy resistance (Zhao et al. 2021). A major limiting factor is the lack of reliable biomarkers for monitoring CICD activity in lung cancer. However, effective biomarkers are required to identify patient subpopulations likely to benefit from CICD-targeted therapies and assess therapeutic efficacy (Renfro et al. 2016; Hu et al. 2022). However, while RIPK1, RIPK3, and MLKL as potential necroptosis makers and GPX4 and SLC7 A11 as potential ferroptosis markers have been used in preclinical studies, they are not broadly applicable in the clinical context (Nicolè et al. 2022). Application to widespread use is inhibited by the variability of their expression across different tumor subtypes, stages, and microenvironments (Zhang et al. 2022 d). Additionally, the spatio-temporal nature of CICD pathways necessitates biomarkers to reflect the time and intensity of activity in pathways (Gim et al. 2024). Real-time monitoring with personalized treatment could be possible with noninvasive diagnostic tools, like liquid biopsies or imaging-based approaches (Adhit et al. 2023).

However, advances in emerging technologies and innovative therapeutic approaches to targeting CICD pathways, coupled with the fact that some of their targets remain druggable, point to the possibility of more effective targeting of CICD pathways in lung cancer. The molecular mechanisms of CICD are being illuminated by advances in omics technologies, including transcriptomics, proteomics, and metabolomics, and new therapeutic targets are being discovered (Dai and Shen 2022). Methods to screen for small molecules that modulate CICD, such as ferroptosis, autophagy, and necroptosis inducers and inhibitors, are greatly accelerated by high-throughput screening (Luo et al. 2024). The discovery expands the therapeutic landscape and opens new possibilities to overcome resistance to conventional treatments (Siqueira-Neto et al. 2023). Preclinical studies have suggested promise in the use of combination therapies using CICD targeting agents with established treatments, such as chemotherapy, radiotherapy, or immune checkpoint inhibitors (Yu et al. 2022). Several preclinical and early-phase clinical studies targeting CICD have been conducted in lung cancer. Ferroptosis inducers, including erastin and RSL3, have shown efficacy in NSCLC models, especially against resistance to EGFR inhibitors. Combining erastin with osimertinib increased tumor regression in xenograft models of resistant lung adenocarcinoma. The proteasome inhibitor bortezomib has also been studied in clinical trials (NCT00431210) for NSCLC. Given its mitochondrial dysfunction and ROS accumulation mechanism, it is a manageable toxicity with preliminary efficacy (Voortman et al. 2007). Hydroxychloroquine (HCQ) inhibitors, as well as autophagy inhibitors, are under investigation in combination with chemotherapy or EGFR-TKIs (e.g. NCT02521051) as treatments to overcome therapy resistance through autophagy inhibition (Liu et al. 2020). Necroptosis-related therapies are being explored in immune-oncology, as RIPK3 expression has been associated with better immune infiltration and outcomes in patients treated by immune checkpoint inhibitors, suggesting they are prognostic and therapeutic targets of necroptosis pathways (Thapa et al. 2024).

In some cases, immune checkpoint inhibitors combined with ferroptosis inducers have shown synergistic effects in which tumor cell killing is improved while increasing anti-tumor immunity (Zheng et al. 2023). Similarly, autophagy modulator medications have made lung cancer cells more sensitive to chemotherapy and radiosensitivity. Such combinations may accelerate therapeutic efficacy or reduce the mechanisms by which single-agent therapies produce resistance (Pritchard et al. 2012). In addition, progress in drug delivery systems allows greater precision and effectiveness of CICD modulation (Adepu and Ramakrishna 2021). CICD-targeting drugs are being developed in nanoparticle-based delivery systems and tumor-targeted therapies that improve the pharmacokinetics and biodistribution of CICD-targeting drugs, decrease off-target effects, and improve therapeutic outcomes (Peng et al. 2024). These platforms facilitate the co-delivery of the CICD inducer and conventional or immune modulation agents to combat lung cancer using a combinatorial strategy (Boone et al. 2022). Recent studies in CICD targeting have highlighted the potential synergistic opportunity of combining them with standard therapies. Specifically, NSCLC cells are sensitive to chemotherapy when they are sensitized with ferroptosis inducers. RSL3, in combination with cisplatin, increased lipid peroxidation and decreased tumor growth in the preclinical model (Yuan et al. 2025). The autophagy inhibitors chloroquine and chloroquine analogues increased radiosensitivity in NSCLC xenograft studies, showing that inhibitors disrupt cytoprotective autophagy in radiotherapy (Saleh et al. 2016). Combining erlotinib with autophagy inhibition using ATG5 siRNA or chloroquine significantly increased cytotoxicity in EGFR mutant NSCLC (Zou et al. 2013). The pairing of CICD modulation with immunotherapy has been very promising as well. Luo et al. showed that a combination of ferroptosis inducers and anti-PD-L1 checkpoint blockade potentiated its anti-tumor immune response and T-cell infiltration in murine lung cancer models (Luo and Xu 2022). These findings support the proposed rationale of combinatorial regimens of CICD modulation and existing strategies in lung cancer.

## Conclusion

In conclusion, this review focuses on key CICD mechanisms, including cell death pathways, necroptosis, ferroptosis, and autophagy-dependent cell death, that can achieve apoptosis-resistant cancer cell elimination. These mechanisms operate independently of caspases and represent unique therapeutic opportunities. RIPK1, RIPK3, and MLKL, coordinated by necroptosis, stimulate anti-tumor immunity. Similar to ferroptosis, drug resistance may be overcome by the process of radical-mediated ferroptosis, especially targeting GPX4 and SLC7 A11. Autophagy has dual therapeutic promise in lung cancer because, although it is a survival mechanism under stress, over-activation of it can result in cell death. CICD has the potential to overcome the limitations of standard lung cancer treatments through anti-apoptotic bypass and modulation of the TME, providing a therapeutic target. For example, the co-administration of ferroptosis inducers with immune checkpoint inhibitors or co-administration of necroptosis modulators with chemotherapies causes synergistic effects in preclinical models that simultaneously increase both cell death and anti-tumor immunity. Similarly, it has been demonstrated that altering autophagy can make cancer cells more sensitive to chemotherapeutics and radiation. While progress has been made, there are still barriers to adapting CICD pathways into clinical practice. However, since specific pathways, like necroptosis and autophagy, operate in dual roles, immoderate regulation can lead to therapeutic benefits over adverse effects. Necroptosis improves immune activation at the expense of potentially proinflammatory effects that inadvertently promote tumor progression. Therefore, targeting autophagy also must be precise enough to regulate the balance to encourage cell death rather than survival. Furthermore, a critical barrier to clinical implementation is the lack of robust biomarkers to monitor CICD activity and predict treatment responses.

## Data Availability

All source data for this work (or generated in this study) are available upon reasonable request.
